# Pathophysiology of dyssynchrony: of squirrels and broken bones

**DOI:** 10.1007/s12471-015-0765-7

**Published:** 2015-12-10

**Authors:** R. F. Wiegerinck, R. Schreurs, F. W. Prinzen

**Affiliations:** Department of Physiology, Cardiovascular Research Institute Maastricht, PO Box 616, 6200 MD Maastricht, The Netherlands

**Keywords:** Heart failure, Cardiac resynchronisation therapy, Ventricular pacing, Dyssynchrony, Protection, Remodelling

## Abstract

The genesis of cardiac resynchronisation therapy (CRT) consists of ‘bedside’ research and ‘bench’ studies that are performed in series with each other. In this field, the bench studies are crucial for understanding the pathophysiology of dyssynchrony and resynchronisation. In a way, CRT started with the insight that abnormal ventricular conduction, as caused by right ventricular pacing, has adverse effects. Out of this research came the ground-breaking insight that ‘simple’ disturbances in impulse conduction, which were initially considered innocent, proved to result in a host of molecular and cellular derangements that lead to a vicious circle of remodelling processes that facilitate the development of heart failure. As a consequence, CRT does not only correct conduction abnormalities, but also improves myocardial properties at many levels. Interestingly, corrections by CRT do not exactly reverse the derangements, induced by dyssynchrony, but also activate novel pathways, a property that may open new avenues for the treatment of heart failure.

## Introduction

Cardiac resynchronisation therapy (CRT) has now been established as a valuable adjunct therapy in patients with heart failure and intraventricular conduction disturbances. CRT is an example where the first evidence of its efficacy was demonstrated in patients, while the underlying pathophysiology was only studied later. In other words: ‘bedside’ research preceded ‘bench’ studies. In subsequent years research moved back and forth between basic research and patient studies. This combination of research has led to a better, although not complete, understanding of the pathophysiology of dyssynchrony and of its treatment by CRT. Out of this research came the ground-breaking insight that ‘simple’ disturbances in impulse conduction, which were initially considered innocent, proved to result in a host of molecular and cellular derangements that lead to a vicious circle of remodelling processes that facilitate the development of heart failure. As a consequence, CRT primarily corrects conduction abnormalities, but secondarily improves myocardial properties at many levels. Interestingly, corrections by CRT are not exactly the reversal of paths induced by dyssynchrony, a property that may open new avenues for treatment of heart failure.

## Electro-mechanics of dyssynchrony

First, it should be acknowledged that the term dyssynchrony is ill defined. Most commonly, it refers to conditions with increased timing differences of either electrical or mechanical activation in the ventricles. Indicators used to this purpose range from the width of the QRS complex in the ECG to the difference in time to peak shortening of strain curves. In most cases electrical dyssynchrony coincides with mechanical dyssynchrony, but mechanical dyssynchrony is found in the absence of a wide QRS complex. The most common causes of ‘true’ dyssynchrony are right ventricular (RV) pacing and left bundle branch block (LBBB). Under these circumstances the normal activation pattern is disturbed because the left ventricle is no longer activated via the left bundle branch and Purkinje fibres. Instead, the electrical activation spreads from the right ventricle through the septum towards the left ventricle. Since activation moving from myocyte to myocyte is much slower than that through the Purkinje system, the left ventricular (LV) free wall—which is the site most remote from the right ventricle—is activated last. Several clinical [1, 2] and pre-clinical [3] electrocardiac mapping studies have shown that the activation in LBBB hearts follows a specific pattern. LV depolarisation moves from the septum in a circumferential and longitudinal direction. However, because conduction often appears slow at the RV-LV junctions, an important contribution of activation comes from the wavefront passing over the apex towards the LV lateral wall (referred to as U-shaped activation pattern). A characteristic feature is also the slow transseptal conduction in LBBB [3], possibly caused by the vertical orientation of the laminar sheets of myocytes in the septum. The prolonged activation of the left ventricle results in a widened QRS complex on the surface electrocardiogram (ECG).

Each action potential triggers the contraction process in myocytes. Indeed, in vivo the sequence of onset and peak shortening is similar to that of the sequence of electrical activation [4]. Recent measurements of electrical activation and peak shortening showed a tight correlation, even in CRT candidates. Interestingly, the slope of the regression line differed between patients, suggesting as yet poorly understood differences between patients [4]. The dyssynchronous electrical activation of the left ventricle causes the early-activated septum to contract against a reduced load, which leads to pre-stretch of the LV free wall [5]. This pre-stretch increases the contractile force of the LV free wall that, in turn, paradoxically stretches the septum later in systole. Both types of systolic stretching can be considered wasted work [5, 6].

This ‘wasted’ work expresses the poorly coordinated contraction, which also results in a poorer pump function, for example expressed as lower rate of rise of the LV pressure (LV dP/dt_max_). Longer lasting LBBB in dogs results in increases of end-diastolic and end-systolic volume and a decrease in ejection fraction (Fig. 1; [7]).

**Figure:**
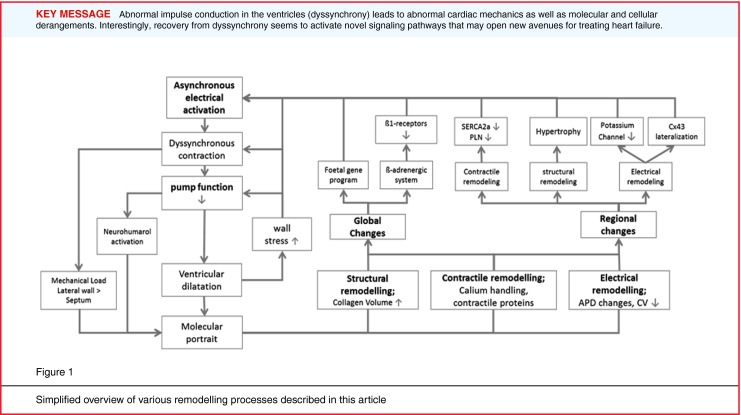

## Remodelling in dyssynchronous hearts: turning the squirrel’s summer coat into a winter coat

The worsening of pump function with longer lasting LBBB is the consequence of a variety of remodelling processes in the myocardium. These processes can partly be attributed to neurohormonal activation, triggered by poor pump function (Fig. 1). A recent human study showed that the baroreflex alters activity within five seconds after changing the activation sequence [8]. Increased sympathetic activation has been demonstrated in dogs during chronic ventricular pacing [9] and greater systemic vascular resistance in patients during ventricular pacing [10]. A recent clinical investigation showed that, in CRT responders, CRT creates a more uniform sympathetic stimulation, as assessed by tracer-uptake studies [11].

Another part of the remodelling process appears to be triggered by the regional differences in mechanical load. As consistently observed in humans and dogs, mechanical load is low in early-activated regions and high in late-activated regions [5, 12, 13]. The latter changes are also reflected by changes in regional blood flow and oxygen consumption [14, 15]. In the long run, this redistribution of mechanical load within the ventricular wall leads to asymmetric hypertrophy [16, 17].

Other factors that contribute to remodelling are the impaired pump function, which leads to ventricular dilatation and thereby increased wall stress, which in turn stimulates the remodelling processes. Moreover, the uncoordinated and dyssynchronous contraction increases the oxygen demand of the myocardium while decreasing the diastolic perfusion time. This combination may lead to lower coronary perfusion reserve and thus higher sensitivity to ischaemia, hibernation and stunning.

A series of studies in the canine model of LBBB, whether or not in combination with tachypacing-induced heart failure [17–19] as well as in the mouse heart after chronic RV pacing [20], have shed light on the changes at the cellular and molecular level in asynchronous hearts. These changes are quite complex, partly mimicking the asymmetry of macroscopic hypertrophy in dyssynchronous hearts. Expression of some genes and proteins is increased or depressed uniformly, while others show regional differences in expression. Examples of uniformly depressed genes and proteins are virtually all potassium channels, several calcium channels and β-adrenergic receptors [18, 19]. Also the foetal gene program and apoptosis-related genes are activated uniformly in dyssynchronous failing hearts. In contrast, the L-type calcium channel and the transient outward potassium current (Ito) show a more pronounced downregulation in the late-activated regions than in the early-activated ones [18]. In addition, in the late-activated LV lateral wall stress response kinases as well as TNFα were upregulated [21] and the gap junction protein connexin 43 showed lateralisation, the latter being associated with slowing down of conduction velocity (Fig. 1; [22]).

Altogether, these global and regional changes lead to a complicated ‘molecular fingerprint’ of remodelling in the dyssynchronous heart. These extensive gene expression changes may be compared with the change of the coat of some animals between the seasons, a change that is driven by temperature and sunlight and is effectuated by alterations in gene expression. The observation that CRT is able to correct the LBBB-induced abnormalities (see below) can then be compared with the recovery of the red squirrel’s beautiful summer coat from the grey winter coat.

## The extensive CRT effects

There are indeed quite some studies supporting the myocardial recovery by CRT, mentioned above. The primary and immediate aim of CRT is to reduce the timing differences in electrical activation as compared with the situation during the conduction abnormality. Importantly, CRT (commonly applied by using biventricular pacing) increases dyssynchrony as compared with normal conduction (narrow QRS complex) and does not benefit, but rather harms, patients with narrow QRS complexes [23, 24]. These results strongly indicate that CRT requires a certain electrical substrate to be effective, the best substrate apparently being LBBB [25].

In connection to the immediate electrical effects of CRT, also LV pump function changes in a beat-to-beat fashion, and remains increased as pacing is continued [17, 26]. The immediate haemodynamic effect of CRT is also used to optimise pacing settings [27]. Interestingly, there is no consistency in the findings regarding the relation between the size of the acute haemodynamic and the long-term echocardiographic changes [28, 29].

**Fig. 2:**
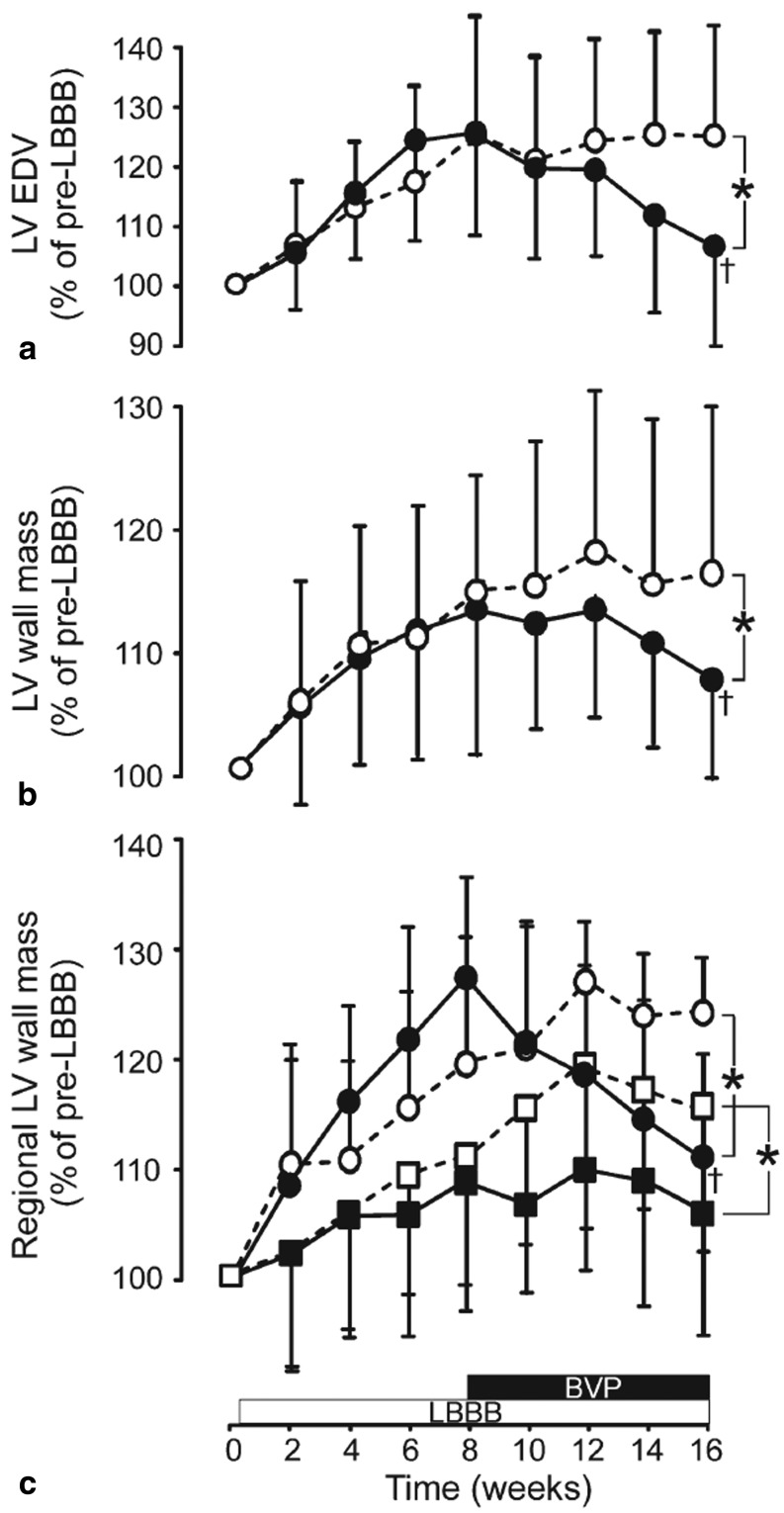
Echocardiographic remodelling in canine LBBB hearts and its reversal upon CRT. Presented are relative changes in (**a**) LV end-diastolic volume (LV EDV), (**b**) total LV wall mass, (**c**) septal (squares) and LV lateral wall (circles) mass in the LBBB (open symbols) and LBBB + CRT groups (closed symbols). *: *p* < 0.05 between LBBB and LBBB + CRT group at 16 weeks. + : *p* < 0.05 between 8 and 16 weeks within the LBBB + CRT group. Values are presented as mean values and SD. *BVP* biventricular pacing, *LBBB* left bundle branch block, *LV* left ventricular (From Vernooy et al. [17])

In experimental LBBB models, where heart rate is kept at a physiological level, chronic application of CRT almost completely recovered the normal geometry and resolved the asymmetric hypertrophy of the LV, as determined by standard echocardiography (Fig. 2; [30]). In the tachycardia-induced LBBB heart failure model, three weeks of rapid biventricular pacing created only a minor improvement in overall pump function, yet considerable molecular changes that did point towards recovery. First of all, CRT normalised the abnormally distributed expression of L-type calcium channel, CaMKinase II, TNFα and p38 [18, 31]. This normalisation process fits with the idea that the local contraction pattern is a determinant of local gene expression. However, also uniformly depressed genes and proteins such as Akt, BAD, Ik, SERCA, β-MHC recovered [31]. Moreover, CRT reduced myocardial catecholamine levels, accompanied by increased expression of β1-adrenergic receptor expression [31]. Yet, the CRT applied at 200 bpm did not result in recovery of Ito and several calcium channels, which were all uniformly depressed during dyssynchronous heart failure [18].

The importance and contribution of remodelling processes in the total benefit of CRT is also illustrated by the common observation that echocardiographic ‘reverse remodelling’ (defined as an increase in LV ejection fraction and/or reduction in LV end-systolic volume) continues to increase over time, even after years of CRT [32]. Electrophysiologically, a human study shows a moderate reduction in intrinsic, non-paced QRS duration after longer lasting CRT [33]. More pronounced electrophysiological changes appear when observing the T-wave during CRT and during temporary halting of CRT in patients. Doing so, a kind of ‘cardiac memory’ develops within approximately two weeks of CRT in patients [34]. These ‘cardiac memory’ effects are also known from temporary halting of RV pacing, so inducing dyssynchrony in patients [35]. Studies in canine hearts using RV pacing have shown that ‘cardiac memory’ involves extensive remodelling at the molecular and cellular level, at least partly related to abnormal myocardial stretch [36]

Molecular, immunohistochemical and cellular studies on myocardial biopsies show significant reduction in collagen deposition and TNFα immunoreaction and reduced cellular apoptotic activity after CRT [37]. Moreover, patients with effective CRT more frequently display chronic enhancement of circulating apelin, a secreted hormone that can block adverse remodelling and has positive inotropic effects [38]. Circulating biomarkers of extracellular matrix remodelling also accompany successful CRT therapy, including decreases in tenascin-C, and metalloproteinases [39]. Chronic CRT also has anti-inflammatory effects, reducing monocyte chemoattractant protein-1, interleukin-8, and interleukin-6 [40].

## Intermittent dyssynchrony: healing the heart like a bone?

The above-mentioned aspects of reversal towards a near-normal myocardial function are to be expected based on the normalisation of contraction patterns. However, there are indications that resynchronisation after a period of dyssynchrony creates an even better condition than a heart that has never experienced dyssynchrony. This is already suggested by the survival curves of LBBB and non-LBBB patients receiving CRT. While the activation patterns during CRT are expected to be the same in LBBB and non-LBBB, absolute survival tends to be better in CRT-treated LBBB patients than in their non-LBBB counterparts [41].

Experimentally, dogs subjected to 6 weeks of atrial tachypacing (synchronous heart failure) were compared with dogs, in which in week 3 and 4 rapid atrial pacing was replaced by rapid RV pacing, thereby inducing temporary dyssynchrony. Myocytes from the latter hearts displayed substantial improvement in function and calcium transients during resting condition as well as during β2-adrenergic stimulation [42] This improvement appeared to relate to higher expression of negative regulators of Gi signalling (yielding Gαs-biased β2-adrenergic stimulation) that was higher than during LBBB and even than during control [42], thus indicating activation of pathways that are specifically activated during resynchronisation after a period of dyssynchrony.

Another example of the potential benefit of temporary dyssynchrony comes from a study involving the trans-catheter aortic valve implantation (TAVI)-induced LBBB in patients with aortic stenosis. While the development of persistent LBBB after TAVI clearly increases the risk of death compared with those not developing LBBB, patients who developed transient LBBB (LBBB disappearing within a year) tended to have a better survival than patients not developing LBBB during TAVI [43, 42].

**Fig. 3:**
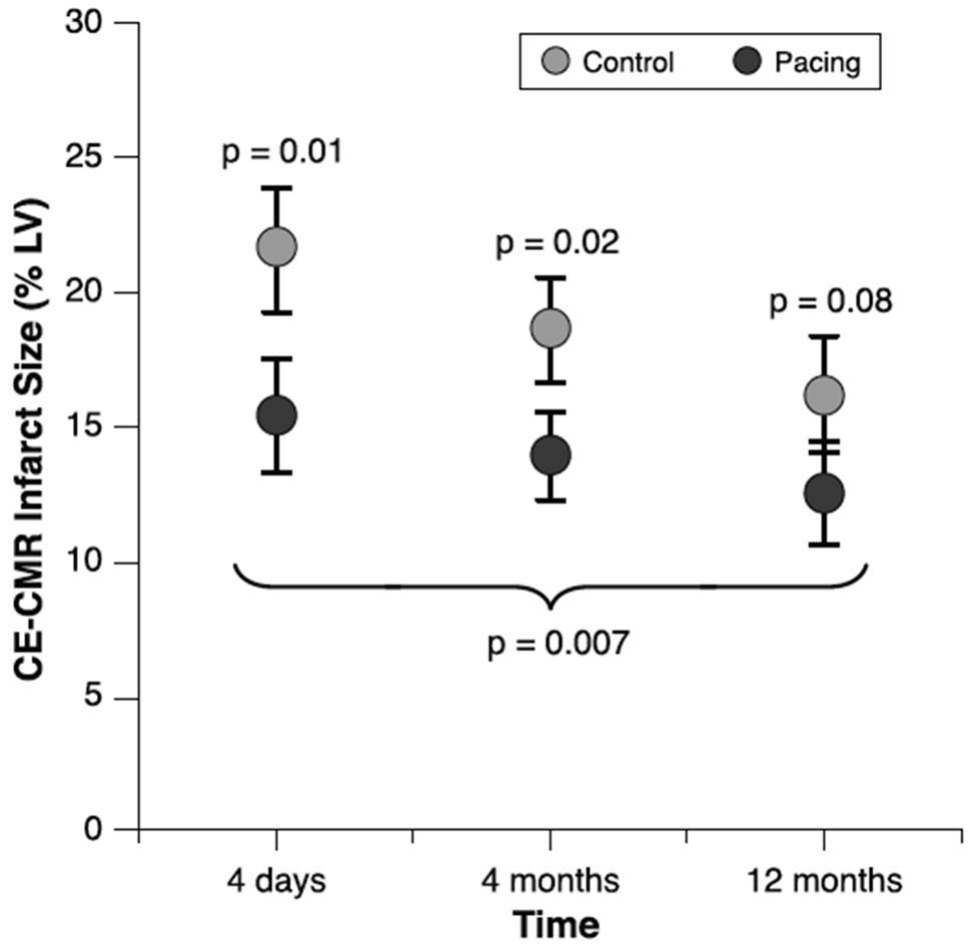
Infarct size (% LV) determined by contrast-enhanced cardiac MRI (CE-CMR) at 4 days (*n* = 48), 4 months (*n* = 44) and 12 months after percutaneous intervention (*n* = 41) in control patients and patients who received transient right ventricular pacing immediately following coronary reperfusion. Data represent adjusted means ± standard error. Data were adjusted for ST-segment deviation, time from symptom onset to hospital admission and affected coronary artery. From Waltenberger et al. [46]

Pushing a potential therapeutic role for intermittent or temporary dyssynchrony further, Vanagt et al. [44] investigated whether the local stretch abnormalities created by dyssynchrony might be cardioprotective. Such protection was demonstrated in rabbit and pig hearts, using brief periods of ventricular pacing before (preconditioning) or after (postconditioning) a period of ischaemia [45]. Pacing postconditioning was also applied in a small study in patients with first myocardial infarction, immediately following percutaneous coronary intervention. Although the study did not meet its primary endpoint (enzyme release), adjusted late-enhancement magnetic resonance images revealed an approximately 25 % reduction in infarct size (Fig. 3; [46]).

The protective effect of pacing postconditioning was not mediated by an ischaemic stimulus, nor by G-protein coupled signalling. The hypothesis that pacing postconditioning is mediated by stretch signalling is supported by the finding that protection could also be induced by intermittently raising the preload in isolated working hearts. Additional proof came from experiments that showed that disruption of microtubules with colchicine and blocking stretch activated channels with gadolinium abrogated the protective effect of pacing postconditioning [47].

Summarising, the aforementioned data indicate that after a period of dyssynchrony, recovery by resynchronisation does not lead to a return exactly back to baseline. Rather, while some derangements appear not amenable, some new pathways (related to sympathetic stimulation and/or myocardial stretch) appear to be activated that lead to a new, supra-normal state. In this regard a comparison may be made with bone healing after a fracture: the fraction heals, and due to the ‘callus’ tissue formed it becomes even stronger than before the fracture. It is premature to make extrapolations to possible applications of intermittent pacing in the field of CRT. However, better understanding the molecular mechanisms of the benefits of intermittent dyssynchrony may lead to novel targets for the treatment of heart failure.

## Conclusions

Evidence is increasing that dyssynchrony can lead to a major and distinctive way of remodelling within the ventricular wall, involving processes that might affect long-term outcome. An important part of that remodelling may be linked to the abnormal contraction pattern rather than to global haemodynamics. Theoretical and practical implications of this hypothesis remain to be proven.

### Funding

This research was performed within the framework of CTMM, the Center for Translational Molecular Medicine (www.ctmm.nl), project COHFAR (grant 01C-203), and supported by the Dutch Heart Foundation.

### Conflicts of interest

FWP received research grants from Medtronic, Boston Scientific, EBR Systems, St. Jude Medical, Sorin, Biological Delivery System (Johnson and Johnson), MSD and Proteus Medical.
